# Comparison of the variability of the annual rates of change in FEV_1_ determined from serial measurements of the pre- versus post-bronchodilator FEV_1_ over 5 years in mild to moderate COPD: Results of the lung health study

**DOI:** 10.1186/1465-9921-13-70

**Published:** 2012-08-15

**Authors:** Donald P Tashkin, He-Jing Wang, David Halpin, Eric C Kleerup, John Connett, Ning Li, Robert Elashoff

**Affiliations:** 1 Departments of Medicine, David Geffen School of Medicine, University of California, Los Angeles, CA, USA; 2 Departments of Biomathematics, David Geffen School of Medicine, University of California, Los Angeles, CA, USA; 3 Royal Devon and Exeter Hospital, Exeter, UK; 4 Department of Biostatistics, University of Minnesota, Minneapolis, MN, USA; 5 David Geffen School of Medicine at UCLA, 10833 Le Conte Ave., Los Angeles, CA, 90095, USA

**Keywords:** FEV_1_ decline, Chronic obstructive pulmonary disease (COPD), Lung health study, Pre-bronchodilator, Post-bronchodilator

## Abstract

**Background:**

The impact of interventions on the progressive course of COPD is currently assessed by the slope of the annual decline in FEV_1_ determined from serial measurements of the post-, in preference to the pre-, bronchodilator FEV_1_. We therefore compared the yearly slope and the variability of the slope of the pre- versus the post-bronchodilator FEV_1_ in men and women with mild to moderate COPD who participated in the 5-year Lung Health Study (LHS).

**Methods:**

Data were analyzed from 4484 of the 5887 LHS participants who had measurements of pre- and post-bronchodilator FEV_1_ at baseline (screening visit 2) and all five annual visits. The annual rate of decline in FEV_1_ (±SE) measured pre- and post-bronchodilator from the first to the fifth annual visit was estimated separately using a random coefficient model adjusted for relevant covariates. Analyses were performed separately within each of the three randomized intervention groups. In addition, individual rates of decline in pre- and post-bronchodilator FEV_1_ were also determined for each participant. Furthermore, sample sizes were estimated for determining the significance of differences in slopes of decline between different interventions using pre- versus post-bronchodilator measurements.

**Results:**

Within each intervention group, mean adjusted and unadjusted slope estimates were slightly higher for the pre- than the post-bronchodilator FEV_1_ (range of differences 2.6-5.2 ml/yr) and the standard errors around these estimates were only minimally higher for the pre- versus the post-bronchodilator FEV_1_ (range 0.05-0.11 ml/yr). Conversely, the standard deviations of the mean FEV_1_ determined at each annual visit were consistently slightly higher (range of differences 0.011 to 0.035 L) for the post- compared to the pre-bronchodilator FEV_1_. Within each group, the proportion of individual participants with a statistically significant slope was similar (varying by only 1.4 to 2.7%) comparing the estimates from the pre- versus the post-bronchodilator FEV_1_. However, sample size estimates were slightly higher when the pre- compared to the post-bronchodilator value was used to determine the significance of specified differences in slopes between interventions.

**Conclusion:**

Serial measurements of the pre-bronchodilator FEV_1_ are generally sufficient for comparing the impact of different interventions on the annual rate of change in FEV_1_.

## Background

The annual rate of change in FEV_1_ has been measured in numerous observational and interventional studies for nearly half a century. In the seminal work of Fletcher and Peto [1], on a cohort of 1136 smokers, an accelerated annual rate of loss of FEV_1_ was observed in a subset of smokers who were believed to be particularly vulnerable to the injurious effects of cigarette smoking leading to the development of chronic obstructive pulmonary disease. This observation established an accelerated annual loss of lung function as a characteristic feature of COPD supporting subsequent definitions of COPD as a progressive disease [2], although recent longitudinal data suggest that COPD is not always progressive [3]. In the aftermath of the Fletcher and Peto publication [1], longitudinal population-based studies have examined the influence of both smoking and other exposures, such as ambient air pollution, on lung function decline, mostly using the FEV_1_ measured without bronchodilator administration [4-6]. Beginning with the first Lung Health Study (LHS I), [7,8], the preferred method of determining the slope of decline in other interventional studies, including trials of inhaled corticosteroids [9-12], N-acetycysteine [13] and long-acting inhaled bronchodilators with or without inhaled corticosteroids [14,15], has relied mainly on the post-bronchodilator FEV_1_ measurement.

The decision to use the slope of the post- as opposed to the pre-bronchodilator FEV_1_ as the primary outcome in LHS I was based, in part, on the assumption that bronchodilator administration would reduce the influence of varying circadian and day-to-day bronchomotor tone on the measurement of FEV_1_[16-18], thereby reducing the variability of the annual slope of FEV_1_ decline, and potentially increasing the power of the study to show a significant difference in the slope of decline in FEV_1_ between the study groups. However, to date, it has not been established that use of the post- compared to the pre-bronchodilator FEV_1_ is associated with a lower variance of the slope of annual change in FEV_1_ in patients with COPD or that there is less month-to-month variability than in the pre-bronchodilator measurements. The objective of the present analysis was to use data from LHS I participants to compare the between-sessions variability of the pre- versus post-bronchodilator FEV_1_ near the beginning of the study and the variance of the annual slope of change in the pre- versus post-bronchodilator FEV_1_ measured over five years. If no difference in the variability of the slope of annual change in FEV_1_ can be discerned between these two methods of measuring the rate of change, then use of only the pre-bronchodilator measurement would simplify longitudinal studies of lung function change without compromising the ability to detect possible differences between different treatment regimens.

## Methods

### Study population and FEV_1_ measurements

**Figure 1:**
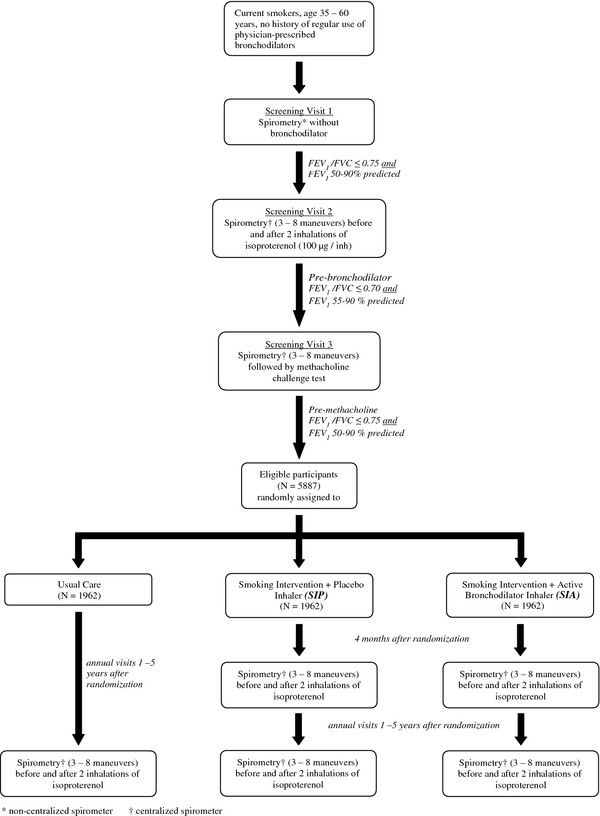
Screening and post-randomization spirometry visits in the Lung Health Study.

LHS I was a ten-center randomized clinical trial of 5,887 middle-aged smokers with the objective of determining whether an intervention program combining intensive smoking cessation counseling and an inhaled anticholinergic bronchodilator could slow the rate of decline in FEV_1_ over a five-year follow-up period during which subjects underwent pre- and post-bronchodilator spirometry annually [7,8]. Entry criteria included a history of current regular smoking, ≥10 pack years of smoking, age 35-60 yrs and the absence of other significant pulmonary or other medical illness, as well as the presence of mild to moderate airflow limitation (see below). A history of asthma treated with regularly scheduled medication was also exclusionary. Potential subjects underwent 3 separate screening visits (Figure 1). At the first screening visit, spirometry was not rigidly controlled, but spirometric methods used at the 2^nd^ and 3^rd^ screening visits and at all visits post-randomization were performed using the same centrally supplied and certified equipment and were rigorously standardized and monitored to maintain suitable quality. At all of these spirometry sessions, three acceptable and two repeatable maneuvers were required from up to eight forced expirations using LHS-specific standards for acceptability and repeatability, as previously described [7,19], and the largest FEV_1_ and FVC values from acceptable and repeatable maneuvers were recorded. Bronchodilator response to two inhalations of isoproterenol (200 μg) from a metered-dose inhaler was determined at the 2^nd^ screening visit and at all subsequent visits, except the 3^rd^ screening visit. At the latter visit, bronchoprovocation with methacholine was performed, the details of which have been published previously [20].

Eligible participants had to have a pre-bronchodilator ratio of FEV_1_ to FVC of ≤0.70 and a pre-bronchodilator FEV_1_ ≤90% predicted and ≥55% predicted [21] at the 2^nd^ screening visit. The “pre-bronchodilator” measurement was defined as the measurement obtained before the administration of a short-acting bronchodilator [isoproterenol] as part of the study, as well as after an adequate washout period following any prior use of bronchodilator medication by participants. Eligible participants were randomized in a 1:2 ratio to receive “usual care” (UC) or “special intervention” (SI). The SI group received a four-month intensive smoking cessation program followed by a five-year relapse prevention program, along with physician advice and nicotine replacement. In addition, participants in the SI group were randomly assigned in a 1:1 ratio and in a double-blind fashion to receive either placebo (SIP group) or ipratropium bromide two inhalations, 18 μg per inhalation (SIA group), which they were instructed to take three times daily for the five-year duration of the study. The UC group received only brief counseling on entry into the study. All subjects were requested to return each year for a total of five post-randomization annual visits during each of which smoking status was determined (and verified by salivary cotinine and end-expired measurements of carbon monoxide) and spirometry was performed both before and after isoproterenol using the same methodology and rigorous standards as applied during the 2^nd^ and 3^rd^ screening visit (Figure 1). In addition, the SI group returned for a four-month post-randomization visit that included pre- and post-bronchodilator spirometry. Informed consent was obtained from all participants at each of the ten LHS clinical centers.

Written informed consent was obtained from all participants originally enrolled in the LHS. The study was approved by the institutional review boards of each of the participating centers and was conducted in accordance with the Declaration of Helsinki and Good Clinical Practice guidelines.

### Statistical analysis

Data from the LHS were reanalyzed to compare the annual FEV_1_ decline measured pre- bronchodilator versus post-bronchodilator for the three study groups (UC, SIP and SIA), separately. Baseline characteristics for the three treatment groups were summarized using descriptive statistics. Among the 5887 subjects in the original study, only the 4484 (~76%) who had measurements at both baseline and all five annual follow-up visits were included in this analysis. Demographic characteristics and mean baseline FEV_1_ values were compared between the included and excluded subgroups. For each included treatment group, the annual rate of decline in FEV_1_ measured pre- and post- bronchodilator from the first to the fifth annual visit was estimated separately using a random coefficient model. Time (year), gender, age, BMI, two-point methacholine concentration-FEV_1_ O’Connor slope [22] and baseline number of cigarettes smoked per day were included in the model as fixed effects; intercept and time (i.e., slope of FEV1) were random effects. The two-point slope was computed as the percent change in FEV_1_ at the highest delivered dose of methacholine from the postdiluent control FEV_1_ divided by the highest concentration of methacholine (in mg/ml) that the subject received. Since, on average, either no change or an increase in FEV_1_ was observed from baseline to the first annual visit, followed by a linear decline in FEV_1_ from annual visits 1 through 5 in the entire LHS 1 population, for the present analysis all slopes were calculated using data from the first through the fifth annual visit. In addition, for each subject individually a linear regression model was used to obtain the annual rate of decline in FEV_1_ for that subject from annual visits 1 through 5. The number of subjects who had a statistically significant individual annual rate of change in FEV_1_ (p < 0.05) and the mean and standard deviation of the slopes for those with and without a significant individual rate of change were tabulated for pre- and post- bronchodilator FEV_1_ separately within each of the three treatment groups.

Based on the estimated slope (annualized change in FEV_1_) and standard deviation determined for the three parallel LHS treatment groups, we performed sample size calculations to determine whether there are differences in the sample sizes needed to demonstrate a significant difference in slopes of FEV1 decline between the UC group and the SIP group, as well as between the UC group and the SIA group, using the pre- versus the post-bronchodilator FEV_1_. For these calculations, we assumed equal sizes for each group, a normal distribution of the annualized change in FEV_1_, a significance level of 0.05 (alpha) and 80% power (beta = 0.2) to detect the observed difference in the annualized change in FEV_1_ between the two study groups in each pair (UC and SIP, and UC and SIA) using a two-sided t-test. Sample sizes were also calculated for the SIA and SIP groups versus a hypothetical comparison study group with an assumed slope difference of 10, 15 and 20 ml/yr, respectively, to investigate possible differences in sample sizes using the pre-bronchodilator vs. the post-bronchodilator FEV1 for determining the slope.

All analyses were performed using SAS software.

## Results

Average baseline characteristics for participants who were included and excluded in the analysis are shown in Table 1 for each study group. Baseline features were modestly but significantly different between the included and excluded participants for the following: in the SIA group, excluded subjects were more often male and non-Caucasian, had a slightly higher BMI and reported smoking more cigarettes/day and a greater number of pack-years; in the SIP group, excluded patients reported smoking more cigarettes/day; and in the UC group, excluded subjects had a lower FEV_1_. No significant differences in baseline characteristics across the subjects in the three treatment groups were noted, except for gender and BMI. The SIA group had more females than the SIP and UC groups (p = 0.009 and 0.006, respectively) and the BMI was slightly lower in the SIA group than the SIP and UC groups (p = 0.0077 and 0.0721, respectively). The latter differences were very small and unlikely to be clinically significant.

**Table 1:** Baseline characteristics of subjects included in versus excluded from the analysis

	**Included in this analysis**	**Study Group**		
		**SIA**	**SIP**	**UC**
**No. of Subjects**	N	457	459	487
	Y	1504	1503	1477
**Subject Characteristics**				
**Gender**				
% Male	N	65.6	64.3	62.6
	Y	59.3	63.9	64.2
		p **=0.0151**	ns	ns
**Age, y**				
Mean ± SD	N	48.7 ± 7.1	48.3 ± 6.9	48.6 ± 7.0
	Y	48.3 ± 6.8	48.6 ± 6.8	48.4 ± 6.8
		ns	ns	ns
**Race**				
% White	N	92.8	94.8	95.3
	Y	96.9	96.1	95.7
		p **=0.0001**	ns	ns
**FEV**_**1**_**, L – pre (S2)**				
Mean ± SD	N	2.62 ± 0.61	2.62 ± 0.58	2.59 ± 0.57
	Y	2.62 ± 0.61	2.65 ± 0.60	2.66 ± 0.60
		ns	ns	p **=0.0178**
**FEV**_**1**_**, L– post (S2)**				
Mean ± SD	N	2.73 ± 0.63	2.73 ± 0.60	2.70 ± 0.59
	Y	2.73 ± 0.64	2.76 ± 0.63	2.78 ± 0.63
		ns	ns	p **=0.0155**
**BMI, kg/ht**^**2**^				
Mean ± SD	N	26.0 ± 4.1	25.7 ± 3.9	25.5 ± 4.1
	Y	25.3 ± 3.9	25.7 ± 3.9	25.6 ± 3.9
		p **=0.002**	ns	ns
**Cigarettes/Day**				
Mean ± SD	N	32.6 ± 13.4	32.8 ± 12.8	31.8 ± 12.6
	Y	30.8 ± 13.1	31.1 ± 12.5	30.9 ± 12.9
		p **=0.006**	p **=0.007**	ns
**Pack/Years**				
Mean ± SD	N	42.6 ± 21.7	41.9 ± 19.9	41.7 ± 20.6
	Y	39.8 ± 19.0	39.9 ± 18.5	40.2 ± 18.3
		p **=0.027**	ns	ns
**O’Connor slope**				
Mean ± SD	N	−14.4 ± 28.1	−12.2 ± 24.1	−12.6 ± 21.6
	Y	−13.2 ± 23.2	−12.0 ± 21.3	−12.9 ± 24.6
		ns	ns	ns

SIA = Special Intervention Anticholinergic; SIP = Special Intervention Placebo; UC = Usual Care; S2 = second screening visit.

Differences for each measure between participants included versus not included in the analysis were tested using the t-test (for age, FEV_1_ and BMI), Chi-square (for gender and race) and Wilcoxon rank sum test (for cigarettes/day, pack-years and O’Connor slope).

**Figure 2:**
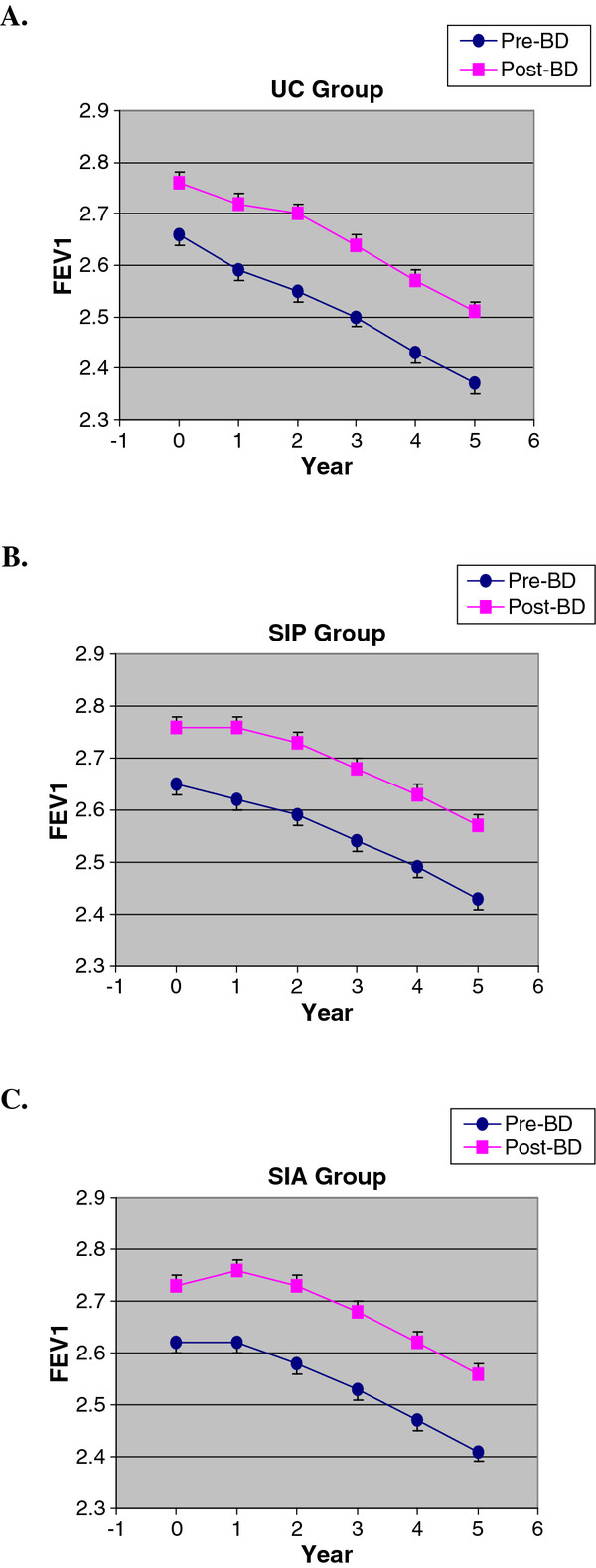
**Mean values of pre- and post-bronchodilator FEV**_**1**_**at screening visit 2 and annual visits 1 through 5 in A) UC group, B) SIP group and C) SIA group.**

The linear slopes (mean and standard deviation) of the pre- and post-bronchodilator FEV_1_ change from annual visits 1 through 5 both unadjusted and adjusted (for age, gender, cigarettes/day and log of the 2-point methacholine concentration-FEV_1_ response slope) are shown for the UC, SIP and SIA groups separately in Table 2. The mean values (± SD) of the pre- and post-bronchodilator FEV_1_ at baseline and each annual visit are shown in Figure 2 and Additional file 1. Regardless of the adjustment for covariates, the mean slopes of both the pre- and post-bronchodilator FEV_1_ are steeper by 12.2-15.5 ml/yr for the UC group compared to both SI groups (Table 2), as previously reported for the post-bronchodilator slope in the entire LHS 1 population [7]. However, within each group, the mean *unadjusted* slope estimates are only slightly higher for the pre- than the post-bronchodilator FEV_1_ (range of differences 2.6-5.2 ml/yr) and the standard errors around these estimates are only minimally higher for the pre- vs. the post-bronchodilator FEV_1_ (range 0.05-0.11 ml/yr). Similarly, the mean *adjusted* slope estimates are slightly higher for the pre- than the post-bronchodilator FEV_1_ in each group (range of differences 2.5-5.1 ml/yr) and the standard errors around these estimates are only minimally higher for the pre- vs. the post-bronchodilator FEV_1_ (range 0.04-0.11 ml/yr). The similarity of the slopes of the pre- vs. post-bronchodilator FEV_1_ from annual visits 1 through 5 is readily discerned by inspection of curves drawn for the mean values of the pre- vs. post-bronchodilator FEV_1_ at each annual visit over this time period (Figure 2). Moreover, the standard deviations of the mean FEV_1_ at each of these time points are very similar on comparison of the pre- and post-bronchodilator values ( Additional file 1).

**Table 2:** **Linear slope estimates (± SE) for the annual change (year 1-5) in pre- and post-bronchodilator FEV**_**1**_**(ml/yr) both unadjusted and adjusted for covariates (age, gender, cigarettes/day and log of the 2-point methacholine-FEV**_**1**_**response slope) by intervention group**

**Group**	**Unadjusted**			**Adjusted**		
	**Slope Estimate ml/yr**	**SE ml/yr**	**p**^**†**^	**Slope Estimate ml/yr**	**SE ml/yr**	**p**^**†**^
UC (N = 1477)						
Pre-bronchodilator	−56.7	1.36		−56.7	1.37	
Post-bronchodilator	−52.8	1.25		−53.1	1.26	
Difference*	3.9	1.07	<0.001	3.6	1.08	<0.001
SIP (N = 1503)						
Pre-bronchodilator	−43.2	1.30		−43.3	1.31	
Post-bronchodilator	−40.6	1.25		−40.8	1.27	
Difference*	2.6	1.07	0.016	2.5	1.09	0.020
SIA (1504)						
Pre-bronchodilator	−42.7	1.36		−42.7	1.38	
Post-bronchodilator	−37.5	1.27		−37.6	1.29	
Difference*	5.2	1.22	<0.001	5.1	1.24	<0.001

UC = Usual Care; SIP = Special Intervention Placebo; SIS = Special Intervention Anticholinergic.

*Difference between the linear slope estimates calculated using the pre- versus post-bronchodilator FEV_1_.

^†^p values refer to the significance of the differences between the slopes determined from the pre- versus post-bronchodilator FEV_1_ within each group (mixed model).

Table 3 shows the percentage of participants in each randomized group who exhibited a statistically significant *individual* slope of pre- and post-bronchodilator FEV_1_ change over annual visits 1 through 5. Within each group, the percentage of individual participants with a statistically significant slope was similar between the slopes estimated from the pre- versus the post-bronchodilator FEV_1_, the differences varying by only 1.4-2.7%. The percentage of UC participants who exhibited a statistically significant slope of both pre- and post-bronchodilator FEV_1_ decline (40.2 and 38.7%, respectively) was significantly higher than the percentage of participants in each SI group (range 25.4-30.5%) who demonstrated a statistically significant slope, most likely attributable to the generally steeper significant slopes in the UC group (range of means 53.5-56.7 ml/yr) than in the two SI groups (range of means 37.5-43.2 ml/yr) as a consequence of the significantly higher rates for sustained quitting in the SI groups than the UC group (7).

**Table 3:** **Proportion of participants (95% CI) in each intervention group with a statistically significant individual annual rate of change in pre- versus post-bronchodilator FEV**_**1**_**from annual visit 1 through 5**

**Intervention Group**	**Proportion with significant change in FEV**_**1**_**(95% CI)**	
	**Pre-bronchodilator FEV**_***1***_	**Post-bronchodilator FEV**_**1**_
UC	40.1 (37.6-42.6)	38.7 (36.2-41.2)
SIP	27.8 (25.6-30.1)	30.5 (28.1-32.8)
SIA	27.0 (24.7-29.2)	25.4 (23.3-27.6)

The differences of the pre- and post-bronchodilator FEV_1_ in the SIP group between screening visit 2 and month 4 reflect the month-to-month variability in FEV_1_ unconfounded by the introduction of maintenance treatment with a bronchodilator ( Additional file2). The mean difference in pre-bronchodilator FEV_1_ from screening visit 2 to month 4 is actually smaller than that for the post-bronchodilator measurements, although the variances are similar.

Using the pre- and post-bronchodilator FEV1, the sample sizes required 1) to demonstrate significant differences between the UC vs. SIA group and the UC vs. SIP group are shown in Table 4 and 2) to demonstrate a significant slope difference of 10, 15 and 20 ml/yr for the SIA and the SIP groups vs. the hypothetical comparison group are shown in Table 5. A smaller N was required to show a difference between the SIP and the UC group using the pre-bronchodilator compared to the post-bronchodilator FEV_1_, while, conversely, a larger N was needed to show a difference between the SIA and UC groups using the pre- compared to the post-BD FEV_1_ (Table 4). These differences in sample size estimates comparing the UC group with each of the SI groups are related to the differences in the means of the slopes (effect size) between the groups, as well as the differences in the SD shown in Table 2. On the other hand, comparison of the sample sizes needed to show statistical significance for specified differences in slope between each SI group and a hypothetical comparison group using pre- vs. post-bronchodilator data showed that slightly larger numbers of subjects would be required using pre- than post-bronchodilator FEV_1_ measurements, especially for relatively small hypothetical differences in slope (Table 5).

**Table 4:** Estimates of sample sizes (N) per group required to demonstrate (A) significant slope differences between the UC vs., separately, the SIA and SIP group using the pre- vs. post-bronchodilator FEV1 and (B) significant slope differences of 10, 15 and 20 ml/yr for the SIA and the SIP group, separately, vs. a hypothetical comparison group using pre- and post-bronchodilator FEV1, respectively

	**SIA vs. UC**	**SIP vs. UC**
Using pre-bronchodilator data	221	227
Using post-bronchodilator data	160	248

**Table 5:** Estimates of sample sizes (N) per group required to demonstrate (A) significant slope differences between the UC vs., separately, the SIA and SIP group using the pre- vs. post-bronchodilator FEV1 and (B) significant slope differences of 10, 15 and 20 ml/yr for the SIA and the SIP group, separately, vs. a hypothetical comparison group using pre- and post-bronchodilator FEV1, respectively

	**Difference of Slope**	**Using pre-BD data**	**Using post-BD data**
SIA vs. comparison group	10	435	382
	15	194	171
	20	110	97
SIP vs. comparison group	10	398	371
	15	178	166
	20	101	94

## Discussion

We found that, for the UC and the two SI groups, both the mean slope and the variance of the slope of the annual change in the pre-bronchodilator FEV_1_ were fairly similar to the slope and variance of the slope determined using the post-bronchodilator FEV_1_ (Table 2), suggesting that the post-bronchodilator measurement offered little advantage over the pre-bronchodilator FEV_1_ for tracking the course of COPD in LHS participants. These findings are consistent with our observation of comparable between-month variability of the pre- and the post-bronchodilator FEV_1_ in the SIP group ( Additional file 1).

Our results indicating that the variability of the slope of annual change in FEV_1_ is not substantially reduced by determining the slope based on the post-bronchodilator compared to the pre-bronchodilator FEV_1_ are further supported by the observation that, within each group, the proportion of participants with a statistically significant *individual* slope of decline in the post-bronchodilator FEV_1_ was similar to the proportion with a statistically significant *individual* slope determined from the pre-bronchodilator FEV_1_ since the statistical significance of the individual slope of FEV_1_ decline is influenced, in large part, by the variance of the slope.

On the other hand, any possible advantage, with respect to savings in time and effort, of restricting the measurement of FEV_1_ to only the pre-bronchodilator value for studies of the impact of an intervention on the annual rate of change in FEV_1_ must be balanced by the comparative size of the sample required, with adequate power, to show a significant difference between the interventions. The sample size is driven by both the effect size and the variance of the annual slope. Therefore, we determined the sample sizes needed to show a significant difference between the UC group and each of the SI groups, as well as to show significant specified differences (10, 15 and 20 ml/yr) between each of the SI groups and a hypothetical comparison group. Inconsistent differences in the required sample size were shown for determining significant differences between the UC group and each of the SI groups using pre- vs. post-bronchodilator data (Table 4). However, in general, a modestly larger sample size was required to demonstrate significance for specified differences in slope between a hypothetical comparison group and each SI group, particularly for relatively small assumed differences in slope (Table 5), thus potentially incurring an additional cost for recruitment of a somewhat larger sample size if only the pre-bronchodilator FEV_1_ were measured.

In studies conducted over approximately the last 25 years comparing the impact of different therapeutic interventions in COPD on the progression of the disease, it became common practice to use the post-bronchodilator, rather than the pre-bronchodilator, FEV_1_ for calculating the annual rate of change as the primary measure of the course of the disease [7,9-15]. The rationale for this practice, is likely to have been based on the belief that the post-bronchodilator value better “standardizes” the FEV_1_ than the pre-bronchodilator value, since the pre-bronchodilator value could be affected by day-to-day and within-day variability in bronchomotor tone, as well as by residual bronchodilation from the last dose of either rescue or maintenance bronchodilator medication if an adequate washout period was not observed. The goal of this “standardization” would be to reduce the variability of the FEV_1_ and thus better estimate the slope of the annual change in FEV_1_, thereby decreasing the sample size required to demonstrate a significant difference in slope between therapeutic interventions.

On the other hand, serial spirometry studies evaluating the time course of FEV_1_ over 24 hrs have failed to show any difference in the circadian pattern of FEV_1_ comparing responses to placebo with those to bronchodilator medication [23,24]. Moreover, the short-term response to a bronchodilator is influenced by several factors [25] in consequence of which the post-bronchodilator increment in FEV_1_ is itself highly variable both within and between patients with COPD [26,27]. One of the factors affecting the acute response to a bronchodilator measured in terms of the absolute improvement in FEV_1_ is the pre-bronchodilator FEV_1_ % predicted, such that, across the spectrum of GOLD stages of severity from moderate to very severe, the magnitude of the FEV_1_ response has been observed to be largest in patients with moderate COPD and smallest in patients with very severe COPD [28,29]. Consequently, as COPD progresses from moderate to very severe airflow obstruction over time, one would expect a progressively smaller absolute increment in FEV_1_ after bronchodilator administration, which could influence the annual slope of decline in the post-bronchodilator FEV_1_. In contrast, patients with relatively mild airflow obstruction, as observed on the baseline visit of the LHS, exhibit a minimal response to a bronchodilator [7,30], in contrast to the much greater response in patients with moderate to severe airflow obstruction, possibly due to the effect of Poisseuille’s Law [31]. Consequently, when such patients progress to a greater degree of airflow obstruction, one would expect a relatively larger acute response to a bronchodilator, as was demonstrated in the continuing and intermittent smokers over the 11 years of follow-up in the LHS [30]. Whether because of these or other factors, the yearly slope of FEV_1_ and the variance of this slope do, in fact, differ between the pre- vs. the post-bronchodilator FEV_1_ has heretofore not been specifically addressed.

This study has several strengths as well as weaknesses. The major strength is the exceptional rigor with which the centralized spirometry assessments were performed and continually monitored for quality control [19], thus minimizing variability due to technical factors. Other strengths include the large number of subjects studied (nearly 6,000, over three-quarters of whom completed all annual visits) and the relatively high representation of females (35%) compared to most other interventional studies in COPD. A weakness is the somewhat limited spectrum of COPD represented by the subjects, all of whom had only mild to moderate airflow obstruction at entry into the study and generally had not been prescribed maintenance bronchodilator or other medication for their COPD, so that our findings might not apply to patients with severe or very severe COPD nor to nonsmokers and those without COPD. Similarly, the average age of the participants (~48 yrs) was much lower than that of COPD patients participating in pharmacotherapeutic trials. The imbalance in some of the baseline characteristics between the ~76% of participants included in the analysis and the remainder who were excluded might be another limitation. To address this limitation, we re-analyzed the data to determine the estimated annual change in FEV_1_ in the total LHS population of 5,887 participants using multiple imputation of the missing data (see Additional file3). The results of this analysis yielded differences between the mean slopes and slope variances determined from the pre- versus the post-bronchodilator FEV_1_ that were very similar to those found when the analysis was restricted only to those who completed all annual visits. Another limitation is that COPD was defined by a pre-bronchodilator ratio of FEV_1_ to FVC of <70%, rather than the currently recommended post-bronchodilator ratio [2]. Consequently, some subjects with fully reversible airflow obstruction were included in the study. There were 503 subjects whose post-bronchodilator FEV_1_ % predicted was 90% or greater, and 1246 whose post-bronchodilator FEV_1_/FVC % was 70% or greater. On the other hand, subjects who were receiving regularly prescribed medication for asthma were excluded.

We conclude that serial measurements of the pre-bronchodilator FEV_1_ appear to be adequate for comparing the impact of different interventions on the annual rate of change in FEV_1_, thus simplifying the design of such longitudinal studies. On the other hand, relying only on the pre-bronchodilator measurement might require a slightly larger sample size to show significant differences between interventions, particularly if relatively small differences in slope are observed. Moreover, measurement of the response to a bronchodilator is important at baseline to exclude the presence of fully reversible airflow obstruction and, in addition, to describe the degree of partial reversibility for descriptive and potential analytic purposes, although the pre-bronchodilator FEV_1_ has been found to be just as accurate as the post-bronchodilator measurement in predicting mortality in the LHS [32]. In addition, if only the pre-bronchodilator measurement is performed over time, care should be taken to ensure that subjects withhold their concomitant bronchodilator medication for a suitable washout period prior to spirometry testing. Furthermore, whether or not post-bronchodilator measurements are also performed, subjects should be studied at approximately the same time of day to minimize variability due to the influence of circadian rhythm.

## Abbreviations

COPD, Chronic obstructive pulmonary disease; FEV1, Forced expired volume in 1 second; UC, Usual Care group; SI, Special Intervention group; SIP, Special Intervention group assigned to placebo; SIA, Special Intervention group assigned to active bronchodilator; S2, Screen 2 visit; M4, Month 4 visit.

## Competing interests

The authors declare that they have no competing interests.

## Authors’ contributions

DT helped design the study, contributed to the collection of data, developed the concept for the statistical analysis, assisted in the interpretation of the results and drafted the manuscript; DH and EK assisted in the interpretation of the results and contributed to the writing of the manuscript; HW and NL carried out the statistical analysis; JC and RE oversaw the statistical analysis and assisted in the interpretation of the results. All authors read and approved the final manuscript.

## Supplementary Material

Additional file 1 **Mean (±SD) pre- and post-bronchodilator FEV**_**1**_**at baseline and each annual visit by intervention group.** Click here for file

Additional file 2 **Mean values ± SD of pre- and post-bronchodilator FEV**_**1**_**at the 2**^**nd**^**screening visit (S2) and the 4 month visit (M4) and the mean M4-S2 differences ± SD in SIP participants (N=1427).** Click here for file

Additional file 3 **Estimates of annual change in FEV**_**1**_(in liters) using multiple imputation of missing data in the entire LHS population*. Click here for file
