# Supplementary material for: Comparison of the variability of the annual rates of change in FEV1 determined from serial measurements of the pre- versus post-bronchodilator FEV1 over 5 years in mild to moderate COPD: Results of the lung health study
Source: Respir Res. 2012 Aug 15;13(1):70. doi: 10.1186/1465-9921-13-70 (PMC3439318; doi:10.1186/1465-9921-13-70)
Supplement: Additional file 1 — Mean (±SD) pre- and post-bronchodilator FEV1at baseline and each annual visit by intervention group. [file 1465-9921-13-70-S1.doc]

**Additional file 1**. Mean (±SD) pre- and post-bronchodilator FEV_1_ at baseline and each annual visit by intervention group

|  | Pre-bronchodilator FEV_1_ | | Post-bronchodilator FEV_1_ | |
| --- | --- | --- | --- | --- |
|  | Mean (L) | SD (L) | Mean (L) | SD (L) |
| Screen 2 visit | | | | |
| UC | 2.663 | 0.598 | 2.775 | 0.633 |
| SIP | 2.648 | 0.596 | 2.761 | 0.627 |
| SIA | 2.618 | 0.608 | 2.727 | 0.640 |
| Year 1 visit | | | | |
| UC | 2.591 | 0.621 | 2.725 | 0.646 |
| SIP | 2.616 | 0.613 | 2.760 | 0.632 |
| SIA | 2.613 | 0.633 | 2.757 | 0.651 |
| Year 2 visit | | | | |
| UC | 2.554 | 0.633 | 2.695 | 0.655 |
| SIP | 2.588 | 0.628 | 2.733 | 0.646 |
| SIA | 2.584 | 0.642 | 2.733 | 0.663 |
| Year 3 visit | | | | |
| UC | 2.497 | 0.640 | 2.635 | 0.664 |
| SIP | 2.541 | 0.633 | 2.680 | 0.648 |
| SIA | 2.530 | 0.654 | 2.677 | 0.674 |
| Year 4 visit | | | | |
| UC | 2.434 | 0.642 | 2.571 | 0.660 |
| SIP | 2.489 | 0.638 | 2.629 | 0.657 |
| SIA | 2.474 | 0.657 | 2.622 | 0.673 |
| Year 5 visit | | | | |
| UC | 2.371 | 0.653 | 2.510 | 0.669 |
| SIP | 2.431 | 0.648 | 2.566 | 0.659 |
| SIA | 2.413 | 0.666 | 2.556 | 0.679 |
