# Supplementary material for: Comparison of the variability of the annual rates of change in FEV1 determined from serial measurements of the pre- versus post-bronchodilator FEV1 over 5 years in mild to moderate COPD: Results of the lung health study
Source: Respir Res. 2012 Aug 15;13(1):70. doi: 10.1186/1465-9921-13-70 (PMC3439318; doi:10.1186/1465-9921-13-70)
Supplement: Additional file 2 — Mean values ± SD of pre- and post-bronchodilator FEV1at the 2ndscreening visit (S2) and the 4 month visit (M4) and the mean M4-S2 differences ± SD in SIP participants (N=1427). [file 1465-9921-13-70-S2.doc]

**Additional file 2.** Mean values ± SD of pre- and post-bronchodilator FEV_1_ at the 2^nd^ screening visit (S2) and the 4 month visit (M4) and the mean M4-S2 differences ± SD in SIP participants (N=1427)

**A. Pre-Bronchodilator measurement**

|  | Mean ± SD | Median | IQR | Min - Max |
| --- | --- | --- | --- | --- |
| S2 | 2.645 ± 0.596 | 2.630 | 2.190 – 3.100 | 1.080 – 4.340 |
| M4 | 2.646 ± 0.606 | 2.640 | 2.190 – 3.090 | 0.910 – 4.510 |
| M4-S2 Difference | 0.001 ± 0.187* | 0.010 | -0.110 – 0.110 | -0.790 – 0.770 |

* p=0.88; paired t test

**B. Post-Bronchodilator measurement**

|  | Mean ± SD | Median | IQR | Min - Max |
| --- | --- | --- | --- | --- |
| S2 | 2.759 ± 0.626 | 2.740 | 2.280 – 3.220 | 1.180 – 4.620 |
| M4 | 2.668 ± 0.608 | 2.650 | 2.220 – 3.110 | 0.950 – 4.570 |
| M4-S2 Difference | -0.091 ± 0.198^†^ | -0.080 | -0.210 – 0.030 | -1.190 – 0.570 |

^†^p=0.0001; paired t test
