# Supplementary material for: Comparison of the variability of the annual rates of change in FEV1 determined from serial measurements of the pre- versus post-bronchodilator FEV1 over 5 years in mild to moderate COPD: Results of the lung health study
Source: Respir Res. 2012 Aug 15;13(1):70. doi: 10.1186/1465-9921-13-70 (PMC3439318; doi:10.1186/1465-9921-13-70)
Supplement: Additional file 3 — Estimates of annual change in FEV1(in liters) using multiple imputation of missing data in the entire LHS population*. [file 1465-9921-13-70-S3.doc]

**Additional file 3**. Estimates of annual change in FEV_1_ (in liters) using multiple imputation of missing data in the entire LHS population*

**1. Without covariates**

SIA-pre

| Parameter | Estimate | Std Error | 95% Confidence Limits | | t Value | Pr > |t| |
| --- | --- | --- | --- | --- | --- | --- |
|
| intercept | 2.645 | 0.014 | 2.6176 | 2.6723 | 189.43 | <.0001 |
| **year** | **-0.0445** | **0.0013** | **-0.047** | **-0.042** | **-34.98** | **<.0001** |

**SIA-post**

| Parameter | Estimate | Std Error | 95% Confidence Limits | | t Value | Pr > |t| |
| --- | --- | --- | --- | --- | --- | --- |
| intercept | 2.76926 | 0.01452 | 2.7408 | 2.7977 | 190.75 | <.0001 |
| **year** | **-0.0406** | **0.00115** | **-0.0429** | **-0.0384** | **-35.34** | **<.0001** |

**SIP-pre**

| Parameter | Estimate | Std Error | 95% Confidence Limits | | t Value | Pr > |t| |
| --- | --- | --- | --- | --- | --- | --- |
| intercept | 2.65184 | 0.0135 | 2.6254 | 2.6783 | 196.49 | <.0001 |
| **year** | **-0.04636** | **0.0012** | **-0.0487** | **-0.044** | **-38.72** | **<.0001** |

**SIP-post**

| Parameter | Estimate | Std Error | 95% Confidence Limits | | t Value | Pr > |t| |
| --- | --- | --- | --- | --- | --- | --- |
| intercept | 2.781354 | 0.014078 | 2.75376 | 2.80895 | 197.57 | <.0001 |
| **year** | **-0.04384** | **0.001196** | **-0.0462** | **-0.0415** | **-36.64** | **<.0001** |

**UC-pre**

| Parameter | Estimate | Std Error | 95% Confidence Limits | | t Value | Pr > |t| |
| --- | --- | --- | --- | --- | --- | --- |
| intercept | 2.640582 | 0.013591 | 2.61394 | 2.66722 | 194.29 | <.0001 |
| **year** | **-0.0581** | **0.001245** | **-0.0605** | **-0.0557** | **-46.66** | **<.0001** |

**UC-post**

| Parameter | Estimate | Std Error | 95% Confidence Limits | | t Value | Pr > |t| |
| --- | --- | --- | --- | --- | --- | --- |
| intercept | 2.763448 | 0.014213 | 2.73559 | 2.79131 | 194.43 | <.0001 |
| **year** | **-0.053757** | **0.00112** | **-0.05595** | **-0.05156** | **-47.98** | **<.0001** |

**2. With covariates**

SIA-pre

| Parameter | Estimate | Std Error | 95% Confidence Limits | | t Value | Pr > |t| |
| --- | --- | --- | --- | --- | --- | --- |
|
| intercept | 4.9664 | 0.0688 | 4.8316 | 5.1012 | 72.22 | <.0001 |
| age | -0.031 | 0.0012 | -0.033 | -0.028 | -24.91 | <.0001 |
| gender | -0.795 | 0.0179 | -0.83 | -0.76 | -44.44 | <.0001 |
| f10cigs | -0.002 | 0.0006 | -0.004 | -0.001 | -3.71 | 0.0002 |
| logoslope | -0.177 | 0.0114 | -0.2 | -0.155 | -15.54 | <.0001 |
| **year** | **-0.044** | **0.0013** | **-0.047** | **-0.042** | **-34.51** | **<.0001** |

**SIA-post**

| Parameter | Estimate | Std Error | 95% Confidence Limits | | t Value | Pr > |t| |
| --- | --- | --- | --- | --- | --- | --- |
| intercept | 5.19539 | 0.0718 | 5.0547 | 5.3361 | 72.36 | <.0001 |
| age | -0.03387 | 0.00128 | -0.0364 | -0.0314 | -26.37 | <.0001 |
| gender | -0.84808 | 0.01865 | -0.8846 | -0.8115 | -45.48 | <.0001 |
| f10cigs | -0.00292 | 0.00068 | -0.0042 | -0.0016 | -4.32 | <.0001 |
| logoslope | -0.14253 | 0.01197 | -0.166 | -0.1191 | -11.91 | <.0001 |
| **year** | **-0.04045** | **0.00116** | **-0.0427** | **-0.0382** | **-34.83** | **<.0001** |

**SIP-pre**

| Parameter | Estimate | Std Error | 95% Confidence Limits | | t Value | Pr > |t| |
| --- | --- | --- | --- | --- | --- | --- |
| intercept | 4.885915 | 0.07023 | 4.74827 | 5.02356 | 69.57 | <.0001 |
| age | -0.02935 | 0.001221 | -0.0317 | -0.027 | -24.03 | <.0001 |
| gender | -0.79406 | 0.018324 | -0.83 | -0.7581 | -43.33 | <.0001 |
| f10cigs | -0.00286 | 0.000672 | -0.0042 | -0.0015 | -4.25 | <.0001 |
| logoslope | -0.17452 | 0.011874 | -0.1978 | -0.1512 | -14.7 | <.0001 |
| **year** | **-0.0463** | **0.001197** | **-0.0486** | **-0.044** | **-38.69** | **<.0001** |

**SIP-post**

| Parameter | Estimate | Std Error | 95% Confidence Limits | | t Value | Pr > |t| |
| --- | --- | --- | --- | --- | --- | --- |
| intercept | 5.144109 | 0.073047 | 5.00094 | 5.28728 | 70.42 | <.0001 |
| age | -0.03286 | 0.001271 | -0.0354 | -0.0304 | -25.85 | <.0001 |
| gender | -0.84694 | 0.019016 | -0.8842 | -0.8097 | -44.54 | <.0001 |
| f10cigs | -0.00313 | 0.000697 | -0.0045 | -0.0018 | -4.49 | <.0001 |
| logoslope | -0.14618 | 0.012332 | -0.1704 | -0.122 | -11.85 | <.0001 |
| **year** | **-0.04386** | **0.001222** | **-0.0463** | **-0.0415** | **-35.9** | **<.0001** |

**UC-pre**

| Parameter | Estimate | Std Error | 95% Confidence Limits | | t Value | Pr > |t| |
| --- | --- | --- | --- | --- | --- | --- |
| intercept | 4.796044 | 0.069465 | 4.65989 | 4.9322 | 69.04 | <.0001 |
| age | -0.02789 | 0.001209 | -0.0303 | -0.0255 | -23.07 | <.0001 |
| gender | -0.7926 | 0.018065 | -0.828 | -0.7572 | -43.88 | <.0001 |
| f10cigs | -0.00218 | 0.000648 | -0.0035 | -0.0009 | -3.36 | 0.0008 |
| logoslope | -0.18004 | 0.011271 | -0.2021 | -0.158 | -15.97 | <.0001 |
| **year** | **-0.05799** | **0.001254** | **-0.0605** | **-0.0555** | **-46.24** | **<.0001** |

**UC-post**

| Parameter | Estimate | Std Error | 95% Confidence Limits | | t Value | Pr > |t| |
| --- | --- | --- | --- | --- | --- | --- |
| intercept | 5.078524 | 0.071805 | 4.93779 | 5.21926 | 70.73 | <.0001 |
| age | -0.03184 | 0.00125 | -0.03429 | -0.02939 | -25.48 | <.0001 |
| gender | -0.853663 | 0.018629 | -0.89018 | -0.81715 | -45.82 | <.0001 |
| f10cigs | -0.002996 | 0.000669 | -0.00431 | -0.00168 | -4.48 | <.0001 |
| logoslope | -0.14798 | 0.011695 | -0.1709 | -0.12506 | -12.65 | <.0001 |
| **year** | **-0.053861** | **0.00113** | **-0.05608** | **-0.05165** | **-47.67** | **<.0001** |

*Missing FEV_1_ measurements were “filled in” by multiple imputation. A Markov Chain Monte Carlo (MCMC) method (Schafer 1997) was used for data imputation due to arbitrary missing patterns. Six imputed datasets were generated. The change in FEV_1_ per year was then estimated within each imputed dataset using linear mixed effects models which assumed random intercept and slope for the underlying trajectory of FEV_1_. The results from the six imputed datasets were combined to produce the final estimates. The analysis was done with and without adjusting for age, gender, cigarettes/day, and log of the 2-point methacholine-FEV_1_ response slope.

Reference:

Schafer, J.L. (1997), *Analysis of Incomplete Multivariate Data*, New York: Chapman and Hall
